# Biomonitoring Study of Deoxynivalenol Exposure in Chinese Inhabitants

**DOI:** 10.3390/ijerph16122169

**Published:** 2019-06-19

**Authors:** Xiaodan Wang, Jiang Liang, Pei Cao, Shuang Zhou, Aibo Wu, Peng Gao, Haibin Xu, Zhaoping Liu, Yunyun Gong

**Affiliations:** 1 Division of Risk Assessment, China National Center for Food Safety Risk Assessment, Beijing 100022, China; wangxiaodan@cfsa.net.cn (X.W.); caopei@cfsa.net.cn (P.C.); gaopeng@cfsa.net.cn (P.G.); hbxu1231602@cfsa.net.cn (H.X.); liuzhaoping@cfsa.net.cn (Z.L.); 2 Key Laboratory of Food Safety Risk Assessment, China National Center for Food Safety Risk Assessment, Beijing 100021, China; zhoush@cfsa.net.cn; 3 Shanghai Institute for Biological Sciences, Chinese Academy of Sciences, Shanghai 200031, China; abwu@sibs.ac.cn; 4 School of Food Science and Nutrition, University of Leeds, Leeds LS2 9JT, UK

**Keywords:** mycotoxins, deoxynivalenol, biomonitoring, biomarker, human urine, exposure assessment

## Abstract

*Objective*: To investigate the levels of a deoxynivalenol (DON) biomarker in the urine of subjects living in two China provinces with different geographic locations and dietary patterns, and estimate their dietary DON exposures and health risks. *Methods*: First morning urine samples were collected on three consecutive days from 599 healthy subjects—301 from Henan province and 298 from Sichuan province—to analyze the total DON concentrations (tDON) after β-glucuronidase hydrolysis using a high-performance liquid chromatography tandem mass spectrometry-based method. The consumption of cereal foods in the previous 24 h before each urine collection was recorded using a duplicate diet method. DON exposure levels were estimated based on the urinary tDON concentrations. *Results*: Total DON were detected in 100% and 92% of the urine samples from Henan and Sichuan, respectively. Mean urinary tDON concentrations were 52.83 ng/mL in Henan subjects and 12.99 ng/mL in Sichuan subjects, respectively. The tDON levels were significantly higher in the urine of Henan subjects than that of the Sichuan subjects (*p* < 0.001). Urinary tDON levels were significantly different among age groups in both areas (Henan: *p* < 0.001; Sichuan: *p* = 0.026) and were highest in adolescents aged 13–17 years, followed by children aged 7–12 years. Based on the DON biomarker and exposure conversion reported by the European Food Safety Authority (EFSA), the mean estimated dietary intakes of DON were 1.82 μg/kg bw/day in Henan subjects and 0.45 μg/kg bw/day in Sichuan subjects. A total of 56% of Henan subjects and 12% of Sichuan subjects were estimated to exceed the PMTDI of 1 μg/kg bw/day. Consistent with urinary tDON levels, the highest estimated dietary DON intakes were also in children and adolescents aged 7–17 years. For all kinds of wheat-based foods except dumplings, the consumptions were significantly higher in Henan than those in Sichuan. The mean consumption of steamed buns was 8.4-fold higher in Henan (70.67 g/d) than that in Sichuan (8.45 g/d). The mean consumption of noodles in Henan (273.91 g/d) was 3.6-fold higher than that in Sichuan (75.87 g/d). *Conclusions*: The levels of urinary DON biomarker and the estimated dietary DON intakes in Henan province were high and concerning, especially for children and adolescents. The overall exposure level of Sichuan inhabitants was low.

## 1. Introduction

Deoxynivalenol (DON), also known as “vomitoxin”, is one of the most commonly occurring mycotoxins in the world, especially in temperate regions [1,2]. It belongs to group B trichothecenes and is produced mainly by *Fusarium graminearum* and *Fusarium culmorum*. Cereal grains including wheat, barley, rye, maize, and oats are the predominant sources of DON exposure [3,4]. The main adverse effects of DON exposure found in animal experiments are gastroenteritis, growth inhibition, immunologic dysregulation, and impairments of reproductive function [2,5,6,7,8,9,10]. There were also a number of reports on human acute intoxications of DON in China and India in the 1980s and 1990s, with cardinal symptoms of nausea, vomiting, diarrhea, abdominal pain, and headaches [11,12]. Although human health effects related to chronic exposure to DON are lacking, given the adverse effects revealed in animal studies, its mechanisms of action, and the ubiquitous contamination of DON, human exposure to DON is regarded as an important food safety issue [13]. To protect human health, the Joint FAO/WHO Expert Committee on Food Additives (JECFA) established a group provisional maximum tolerable daily intake (PMTDI) for DON and its acetylated derivatives (3-Ac-DON and 15-Ac-DON) of 1 μg/kg bw/day [11]. In response, many countries including the European Union, Norway, Japan, Pakistan, and China performed DON exposure assessment in corresponding populations [14,15,16,17,18].

The traditional method to assess the exposure to DON is based on representative food contamination data combined with food consumption data. Similar to all mycotoxins, the distribution of DON in foods exhibits high heterogeneity, and is thus subject to poor food sampling representativeness [19]. The inaccuracy in food consumption measurement and the multiple food sources of DON in diets contribute to the difficulty for reliable exposure assessment. With the understanding of DON metabolism in vivo and the development of analytical methods of DON biomarkers, assessment of DON exposure based on biomarkers is considered to be an advanced approach, and has been used in an increasing number of studies [20,21,22,23] because it avoids the influence by the heterogeneous distribution of DON in food samples and the variation of DON concentration in foods due to food processing [19]. Many studies conducted in the past decade have demonstrated that the main fraction of DON ingested is excreted in the urine in its unmetabolized form (free DON) and DON-glucuronides (DON-GlcA), including DON-15-glucuronide (DON-15-GlcA) and DON-3-glucuronide (DON-3-GlcA) [19,24,25,26]. The sum of free DON and DON-GlcA in human urine, i.e., total DON (tDON), was well correlated with the consumption of DON-contaminated cereals, and is thus considered an appropriate biomarker of DON exposure for risk assessment [27,28,29,30]. In addition to DON, deepoxy-deoxynivalenol (DOM-1) and its glucuronides are also frequent metabolites excreted in urine, especially in children [31]. However, the detection rate and content level of DOM-1 are much lower than those of DON [12,23]. Thus, DOM-1 was not a preferred biomarker of exposure, especially in large human epidemiological studies.

China is a country that is mainly located in a temperate zone, and the grain-based dietary pattern makes Chinese residents at relatively high risk due to DON exposure. Results of dietary exposure assessment indicated high exposure levels in some Chinese populations [18]. However, to date, few biomonitoring studies have been conducted in China, and there are very limited data on levels of DON biomarkers in Chinese people. To fill in the data gap, we conducted a survey in two Chinese provinces located in the north (Henan province) and south (Sichuan province), respectively, with very different consumption patterns of staple foods (Henan inhabitants consume wheat as their staple food, whilst those in Sichuan consume rice as their staple food). The two selected provinces can represent two typical DON exposure scenarios in China. The aim of this study was to investigate the levels of DON biomarker in the urine of subjects living in the two provinces with different geographic locations and dietary patterns, and estimate their dietary DON exposures as well as the corresponding health risks.

## 2. Materials and Methods

### 2.1. Subjects Recruitment

Subjects aged above 1 year were randomly recruited from several villages and communities in Henan province (*n* = 301) and Sichuan province (*n* = 298) during November to December 2015. All the subjects included had been living in the surveyed areas for at least 1 year, and were apparently healthy. Subjects with diabetes were excluded for the possible change of cereal consumption habits.

This study was approved by the ethics committee of China National Center for Food Safety Risk Assessment (identification code: 2016030063). Informed written consents were obtained from all the subjects or their statutory guardians when juveniles were considered.

### 2.2. Data and Sample Collection

Approximate 40 mL of first morning urine samples were collected from each subject and were immediately frozen at −20 °C on three consecutive days (two working days and a weekend day). The consumption of cereal-based foods in ready-to-eat state in the previous 24 h before each urine sample collection were recorded using a duplicate diet method. Demographic and anthropometric data including age, gender, and body weight were recorded in the meantime. All the field worker investigators were trained before the survey started.

### 2.3. Laboratory Analysis

The three urine samples collected from each subject were thawed and mixed by equal proportion. All the mixtures were centrifuged (5000× *g*, 15 min) and then processed with β-glucuronidase digestion to release DON from its glucosiduronide conjugates. The tDON concentration in each urine sample after enzyme digestion was analyzed as the exposure biomarker using a high-performance liquid chromatography tandem mass spectrometry-based method described by Brera et al. [25] and Deng et al. [32]. The limit of detection (LOD) and limit of quantitation (LOQ) for tDON in urine were 0.5 ng/mL and 1.0 ng/mL, respectively.

### 2.4. Estimation of Dietary DON Intakes

Dietary intake of DON was estimated based on urinary tDON concentration using the following formula reported by European Food Safety Authority (EFSA) and others [12,32,33,34] for calculation.

(1) $$\mathrm{Dietary}\;\mathrm{DON}\;\mathrm{intake}\;\left(\mu g⁄\mathrm{kg}\;\mathrm{bw}/\mathrm{day}\right)=\frac{\mathrm{Urinary}\;\mathrm{tDON}\;\left(\mu g⁄L\right)\times 24\;h\;\mathrm{urine}\;\mathrm{volume}\left(L\right)}{\mathrm{Excretion}\;\mathrm{rate}\;\left(0.7\right)\times \mathrm{Body}\;\mathrm{weight}\;\left(\mathrm{kg}\right)}$$

The average excretion rate was assumed to be 70% [12], and the 24-h urine volume was assumed to be 0.5 L for children aged 1–6 years, 1.0 L for children and adolescents aged 7–17 years, and 1.5 L for adults according to the published data [12,23,33,35]. The estimated dietary DON intakes were compared to the PMTDI of 1 μg/kg bw/day to assess the risk of DON exposure.

### 2.5. Statistical Analysis

SAS 9.4 (SAS Institute Inc., Cary, NC, USA) was used for statistical analysis. DON concentrations of unquantified samples were set as half value of the LOD. Data of urinary tDON concentrations and estimated dietary DON intakes didn’t fit normal distribution with or without logarithmic transformation, and were described by means, standard deviations, and quantiles. Differences in the above data between the two surveyed areas or genders were analyzed using a Wilcoxon independent sample rank sum test. Differences in urinary tDON levels and estimated dietary DON intakes among age groups were analyzed using the Kruskal–Wallis H-test. Findings were considered statistically significant at *p-*value < 0.05.

## 3. Results

### 3.1. Characteristics of Subjects

A total of 599 subjects, 301 from Henan province (107 males and 194 females) and 298 from Sichuan province (146 males and 152 females), were included in the analysis. The mean body weight was 61.53 kg for male subjects and 51.41 kg for female subjects. All the subjects were allocated into five age groups, i.e., 1–6 years, 7–12 years, 13–17 years, 18–59 years, and ≥60 years. The number of subjects in each age group were 65, 61, 64, 260, and 149, and the mean body weight were 18.03 kg, 35.08 kg, 53.93 kg, 66.48 kg, and 62.47 kg, respectively.

### 3.2. Urinary tDON Concentrations of the Subjects

Total DON was detected in most of the urine samples after enzyme hydrolysis with a positive rate of 100% in samples from Henan subjects and 91.9% in Sichuan subjects’ samples. Descriptive data of urinary tDON concentrations by area, age, and gender were presented in Table 1.

Mean urinary tDON concentrations were 52.83 ng/mL in Henan subjects and 12.99 ng/mL in Sichuan subjects, respectively. The median and maximum values were 34.33 ng/mL and 335.84 ng/mL in Henan whilst 6.02 ng/mL and 149.62 ng/mL in Sichuan, respectively. Total DON levels were significantly higher in the urine of Henan subjects than those of Sichuan subjects (*p* < 0.001). Between genders, urinary tDON levels were significantly higher in female subjects than those in male subjects in Henan (*p* < 0.001), and the mean values were 62.26 ng/mL and 35.72 ng/mL, respectively. However, in Sichuan province, urinary tDON levels were not significantly different between genders (*p* = 0.325), and the mean values were 13.71 ng/mL in male subjects and 12.31 ng/mL in female subjects.

Among age groups, urinary tDON levels were significantly different in both areas (Henan: *p* < 0.001; Sichuan: *p* = 0.026) with the mean values of 55.71 ng/mL, 78.36 ng/mL, 100.18 ng/mL, 47.89 ng/mL, and 26.54 ng/mL in Henan subjects aged 1–6 years, 7–12 years, 13–17 years, 18–59 years, and ≥60 years, and 10.13 ng/mL, 15.86 ng/mL, 24.65 ng/mL, 12.11 ng/mL, and 10.20 ng/mL in corresponding age groups in Sichuan. Urinary tDON concentrations were highest in subjects aged 13–17 years in both areas, followed by children aged 7–12 years, whereas the lowest levels were in the elderly (≥60 years). Compared with adults (18–59 years), the difference was statistically significant for 7–12 years (*p* = 0.010), 13–17 years (*p* < 0.001), and ≥60 years (*p* < 0.001) in Henan, whilst in Sichuan, the difference was significant for 13–17 years (*p* = 0.013) only. Urinary tDON concentrations were significantly higher in Henan subjects than those reported in Sichuan subjects in all age groups (all *p* values < 0.001).

### 3.3. Estimated Dietary DON Intakes

The estimated dietary DON intakes based on urinary tDON concentrations and the results of risk assessment were provided in Table 2. The mean and median estimated dietary intakes of DON were 1.82 μg/kg bw/day and 1.17 μg/kg bw/day in Henan subjects and 0.45 μg/kg and 0.21 μg/kg bw/day in Sichuan subjects. A total of 55.8% of Henan subjects and 12.1% of Sichuan subjects were estimated to exceed the PMTDI of 1 μg/kg bw/day. Consistent with urinary tDON levels, the estimated dietary DON intakes were significantly higher in Henan than those in Sichuan (*p* < 0.001) and were also significantly different among age groups (*p* < 0.001). Mean estimated dietary intakes of DON were 2.22 μg/kg bw/day, 3.80 μg/kg bw/day, 2.64 μg/kg bw/day, 1.52 μg/kg bw/day, and 0.90 μg/kg bw/day in Henan subjects aged 1–6 years, 7–12 years, 13–17 years, 18–59 years, and ≥60 years, whilst 0.40 μg/kg bw/day, 0.63 μg/kg bw/day, 0.67 μg/kg bw/day, 0.41 μg/kg bw/day, and 0.39 μg/kg bw/day in corresponding age groups in Sichuan. There was a significant difference in estimated dietary DON intakes among age groups in Henan (*p* < 0.001), with the highest intakes in children and adolescents aged 7–17 years, whilst the lowest intakes were in subjects aged 60 years and over. Compared with adults (18–59 years), the difference was statistically significant for 7–12 years, 13–17 years, and ≥60 years (all *p* values < 0.001) in Henan. However, for Sichuan subjects, the estimated dietary DON intakes were not significantly different among age groups. 81.6% (84/103) of juveniles (<18 years) in Henan, and 16.1% (14/87) of juveniles in Sichuan were estimated to exceed the PMTDI.

### 3.4. Consumption of Cereal-Based Foods

Table 3 presents the consumptions of the main kinds of cereal-based foods in the two surveyed areas, including steamed buns, noodles, steamed rice, rice porridge, pancakes, steamed stuffed buns, and dumplings. The top three most frequently consumed cereal-based foods in Henan province were noodles, steamed rice, and steamed buns, and the percentage of eaters were 96.01% (289/301), 86.38% (260/301), and 72.43% (218/301) in sequence. The most frequently consumed cereal-based foods were steamed rice, rice porridge, and noodles in Sichuan with the percentage of eaters at 94.97% (283/298), 36.24% (108/298), and 22.82% (68/298), respectively. For all kinds of wheat-based foods except dumplings, the consumptions were significantly higher in Henan than those in Sichuan (steamed buns, noodles, and pancakes: *p* < 0.001; steamed stuffed buns: *p* = 0.001). Among wheat-based foods, steamed buns and noodles were the primary kinds with the highest frequency of consumption in both areas. The mean consumption of steamed buns was 8.4-fold higher in Henan (70.67 g/d) than that in Sichuan (8.45 g/d). The mean consumption of noodles in Henan (273.91 g/d) was 3.6-fold higher than that in Sichuan (75.87 g/d). For rice-based foods, the consumptions of steamed rice were much higher in Sichuan than those in Henan (*p* < 0.001), with the mean consumptions of 561.20 g/d and 120.33 g/d, respectively. The consumptions of rice porridge were not significantly different between the two areas (*p* = 0.267).

## 4. Discussion

DON exposure varies considerably with geographic location, climatic conditions, economic development, as well as agricultural infrastructure, policy, and food adequacy [1]. The two surveyed areas in this study, Henan province and Sichuan province, were located in separate parts of China with discrepant climate and dietary patterns so that there might be differences in DON exposure levels between the two areas. The assessment performed in this study confirmed the wide difference in urinary tDON levels and estimated dietary DON exposure between subjects in Henan and Sichuan. Overall, the estimated DON exposures of Henan subjects were high, with a mean exposure of 1.82 μg/kg bw/day and more than half (56%) of individuals exceeding the PMTDI, whereas the exposures of Sichuan subjects were low, with a mean exposure of 0.45 μg/kg bw/day and 12% of individuals exceeding the PMTDI. Other studies conducted in Chinese populations also revealed interregional differences in DON exposure. Meky et al. [36] investigated the urinary DON levels in Linxian county, Henan province, and Gejiu city, Yunnan province (in southern China) in a small-scale study (*n* = 15). The mean urinary DON level was found to be 37 ng/mL in Henan subjects, which was lower than the mean value of Henan subjects (52.83 ng/mL) obtained in our study. The mean urinary DON level was 12 ng/mL in Yunnan subjects in Meky’s study, which was close to the mean value of Sichuan subjects (12.99 ng/mL) in our study. Assessments performed based on food analysis nationwide showed that the dietary exposures to DON were higher in northern China than those in southern China with the mean exposures about 1.15 μg/kg bw/day and 0.41 μg/kg bw/day, respectively [18], which was consistent with the results of this study, and thus manifested that the methods used in our study to estimate dietary DON exposure based on urinary DON biomarker were reliable.

DON mainly contaminates cereal crops including wheat, corn, barley, rye, and oats, and humans are exposed to DON predominantly by consuming cereal-based foods [4,11]. It has been demonstrated that urinary DON concentrations were well correlated with the consumptions of DON-contaminated cereals [27]. In this study, consumptions of cereal-based foods by the subjects were analyzed, and great differences in cereal consumption patterns were recognized between the two areas. The consumptions of wheat-based foods in Henan were significantly higher than those in Sichuan, while the consumptions of rice-based foods were on the contrary, which was consistent with other studies [37]. Risk assessment performed in the Chinese population indicated that wheat-based food products were the main source of DON exposure [18]. Thus, it is believed that the diversity in the consumption of wheat-based foods was an important reason for the difference in concentrations of the urinary DON biomarker as well as estimated dietary DON exposures between the two areas. Besides, many studies reported that Henan was a province with a high incidence of Fusarium head blight and high levels of DON contamination [38,39]_ENREF_40. Therefore, different DON contamination levels in foods might be another reason for the interregional difference in DON exposure.

There are some other studies reporting the levels of DON biomarkers in human urine around the world. Several studies were conducted in United Kingdom (UK) adults, and the mean urinary tDON levels ranged from 7.1 to 17.8 ng/mL [27,28,29,40], which was approximate to the mean level of Sichuan adults in this study (18–59 years: 12.11 ng/mL). Wallin et al. [21,41] analyzed the urinary tDON levels in Sweden adults, and a median level of 2.9 ng/mL and a mean level of 4.4 ng/mL were obtained, which were even lower than the Sichuan adults in our study (median: 5.4 ng/mL; mean: 12.11 ng/mL). The results of other studies in European sites include: the median urinary tDON level of 76 France subjects, which was 6.8 ng/ml [42]; the mean urinary tDON level of 52 Italy subjects aged 3–85 years, which was 11.9 ng/ml [34]; and the mean urinary tDON in Germany was reported to be 13.2 ng/mL [33]. Compared to the above studies, the urinary tDON levels of Sichuan subjects reported in our study were at the similar level with the European populations, while the urinary tDON levels of Henan subjects (median value: 34.33 ng/mL; mean value: 52.83 ng/mL) were much higher than those of the Europeans. However, there have been studies conducted in Belgium and Croatia that reported even higher urinary tDON concentrations than those in Henan, with mean values of approximately 65.2 to 167.5 ng/ml [22,43]. Studies carried out in regions other than Europe also revealed the variability in levels of urinary DON biomarkers in different populations. The mean level of urinary tDON in Haiti subjects was reported to be 20.2 ng/ml [33]. Bangladesh adults and pregnant women were exposed to much lower levels of DON, with mean urinary tDON concentrations of 0.17 and 0.86 ng/mL, respectively, as reported by Ali et al. [20,44].

Urinary tDON levels and estimated dietary DON exposures were highest in children and adolescents in this study. This was consistent with the data obtained from the UK, Italian, and Norwegian populations reported in the EFSA’s report [12]. Papageorgiou’s study [23] also presented a relatively higher level of urinary tDON in UK children and adolescents (mean concentrations were approximately 20.6 to 38.2 ng/mL in children aged 3–9 years and approximately 20.6 to 28.8 ng/mL in adolescents aged 10–17 years) compared with that in the UK adults reported above. Mitropoulou’s study [31] reported a higher positive rate of urinary DON in children (94%) than in adults (63%). Makri et al. [45] reviewed children’s susceptibility to chemicals, and indicated that their greater dietary intake on a weight basis compared with adults resulted in higher exposure to chemicals in children. Thus, the reason for higher DON exposure in children in our study was believed to be their higher cereal food intake per kg of body weight. In addition to the higher exposure level, children and adolescents are considered to be more sensitive to the adverse effects caused by DON due to the ongoing development of their body system and limited ability for detoxification compared with adults [8,45]. Therefore, DON exposure in children and adolescents deserves attention from the government and the public, especially in Henan province, where 81.6% of the juvenile subjects were estimated to exceed the PMTDI set by JECFA.

The limitations of this study include the lack of 24-h urine collection; as a result, the estimation of dietary DON intake was not accurate. Furthermore, the carry over of DON from dietary intakes to urine may be estimated if the DON contamination data in cereal-based foods consumed by the subjects were obtained. There is also a certain degree of uncertainty in the estimation of dietary DON exposure due to the individual variation of DON excretion rate and the different calculating methods.

## 5. Conclusions

In summary, this study revealed high levels of the urinary DON biomarker and estimated dietary DON intakes in Henan, especially in children and adolescents. This raised serious concerns regarding the high risk of DON exposure in Henan province. Although a small number of subjects in Sichuan exceeded the PMTDI, the overall exposure level of Sichuan inhabitants was low. To get a full picture of the urinary DON biomarker levels of Chinese residents and find out other high-risk populations, there is an urgent need for larger-scale studies in different regions of China.

## Figures and Tables

**Table 1:** Urinary total deoxynivalenol (tDON) concentrations of the subjects.

Age/Area		*n*	Total DON (ng/mL)						*p*
			Mean ± SD	P_25_	P_50_	P_75_	P_90_	Maximum	
1–6 years	Henan	35	55.71 ± 48.55	19.76	41.99	82.58	100.16	224.14	
	Sichuan	30	10.13 ± 12.73	2.03	5.43	12.30	27.90	56.30	<0.001
7–12 years *	Henan	32	78.36 ± 60.94	42.25	53.24	122.77	159.83	240.87	
	Sichuan	29	15.86 ± 29.24	2.83	6.10	14.60	31.09	148.00	<0.001
13–17 years *^#^	Henan	36	100.18 ± 76.35	47.05	81.17	140.25	214.80	335.84	
	Sichuan	28	24.65 ± 25.04	7.85	12.95	33.15	69.20	85.30	<0.001
18–59 years	Henan	121	47.89 ± 47.22	16.58	32.54	63.68	102.94	228.18	
	Sichuan	139	12.11 ± 20.47	2.07	5.40	11.70	33.00	149.62	<0.001
≥60 years *	Henan	77	26.54 ± 38.75	9.96	14.74	30.86	47.81	247.08	
	Sichuan	72	10.2 ± 12.69	1.38	5.76	13.45	24.40	69.98	<0.001
Henan	Male	107	35.72 ± 37.63	12.24	24.09	42.82	82.58	228.18	
	Female	194	62.26 ± 61.94	16.60	43.15	86.62	152.16	335.84	<0.001
	Total	301	52.83 ± 55.95	14.84	34.33	68.25	129.31	335.84	
Sichuan	Male	146	13.71 ± 24.41	1.85	6.33	12.82	32.30	149.62	
	Female	152	12.31 ± 14.84	2.61	5.76	17.55	30.70	69.41	0.325
	Total	298	12.99 ± 20.08	2.10	6.02	14.92	31.50	149.62	

For each subject, the tDON concentration was derived from a mixture of three urine samples collected on three consecutive days. * Urinary tDON levels were significantly different (*p* < 0.05) compared with 18–59 years group in Henan subjects. ^#^ Urinary tDON levels were significantly different (*p* < 0.05) compared with 18–59 years group in Sichuan subjects.

**Table 2:** Estimated dietary DON intakes of the subjects.

Age Group	Henan					Sichuan					*p*
	*n*	Mean ± SD	Median	P_90_	% Exceeding PMTDI	*n*	Mean ± SD	Median	P_90_	% Exceeding PMTDI	
1–6 years	35	2.22 ± 1.68	1.83	4.31	74.29	30	0.40 ± 0.51	0.24	1.00	10.00	<0.001
7–12 years *	32	3.80 ± 3.73	2.23	9.93	87.50	29	0.63 ± 0.96	0.25	1.70	20.69	<0.001
13–17 years *	36	2.64 ± 1.93	2.01	5.93	83.33	28	0.67 ± 0.71	0.36	2.08	17.86	<0.001
18–59 years	121	1.52 ± 1.60	1.06	3.15	52.07	139	0.41 ± 0.64	0.16	1.14	11.51	<0.001
≥60 years *	77	0.90 ± 1.31	0.46	1.73	27.27	72	0.39 ± 0.52	0.22	0.91	8.33	<0.001
Total	301	1.82 ± 2.10	1.17	4.28	55.81	298	0.45 ± 0.65	0.21	1.14	12.08	<0.001

PMTDI: provisional maximum tolerable daily intake. For each subject, the food consumption was the mean value of food consumptions on three consecutive days. * Estimated dietary DON intakes were significantly different (*p* < 0.05) compared with 18–59 years group in Henan subjects.

**Table 3:** Consumption of the cereal-based foods.

Food Category	Henan			Sichuan			Z	*p*
	Eaters% (Eaters/Respondents)	Mean ± SD	Median	Eaters% (Eaters/Respondents)	Mean ± SD	Median		
Wheat-derived foods								
Steamed buns	72.43 (218/301)	70.76 ± 79.48	47.63	6.38 (19/298)	8.45 ± 35.95	0	15.45	<0.001
Noodles	96.01 (289/301)	273.91 ± 175.67	241.67	22.82 (68/298)	75.87 ± 176.53	0	15.57	<0.001
Pancakes	12.62 (38/301)	6.60 ± 22.18	0	1.34 (4/298)	1.14 ± 10.59	0	5.37	<0.001
Steamed stuffed bun	11.30 (34/301)	18.19 ± 64.78	0	4.36 (13/298)	3.42 ± 21.17	0	3.25	0.001
Dumplings	5.98 (18/301)	9.25 ± 41.29	0	2.68 (8/298)	6.49 ± 42.12	0	1.923	0.054
Rice-derived foods								
Steamed rice	86.38 (260/301)	120.33 ± 93.30	106.27	94.97 (283/298)	560.74 ± 421.57	494.3	–17.34	<0.001
Rice porridge	40.53 (122/301)	98.33 ± 164.23	0	36.24 (108/298)	161.11 ± 273.75	0	–0.95	0.343

The consumption of all foods was weighed at the ready-to-eat state after cooking with water included.
